# Effects of Fruiting Load on Endogenous Hormones in the Aril of Longan Fruit and Leaflet of Fruiting Branches at the Mature Stage

**DOI:** 10.3390/plants15030353

**Published:** 2026-01-23

**Authors:** Junbin Wei, Shilian Huang, Jingyi Li, Dongmei Han, Tao Luo, Jianguang Li, Zhenxian Wu, Dongliang Guo, Xinmin Lv, Yanan Tian

**Affiliations:** 1 Institute of Fruit Tree Research, Guangdong Academy of Agricultural Sciences/Key Laboratory of South Subtropical Fruit Biology and Genetic Resource Utilization, Ministry of Agriculture and Rural Affairs/Guangdong Provincial Key Laboratory of Science and Technology Research on Fruit Tree, Guangzhou 510640, China; 2 College of Horticulture, South China Agricultural University/Guangdong Provincial Key Lab for Postharvest Science of Fruits and Vegetables/Engineering Research Center of Southern Horticultural Products Preservation, Ministry of Education, Guangzhou 510642, China; 3 Shanghai Key Laboratory of Plant Molecular Sciences, College of Life Sciences, Shanghai Normal University, Shanghai 200234, China

**Keywords:** longan, fruiting load, endogenous hormone, growth and fruiting, fruit quality

## Abstract

Longan (*Dimocarpus longan* Lour.) exhibits vigorous vegetative growth and strong fruit setting ability but suffers from alternate bearing. The role of endogenous hormones in mediating the effects of fruiting load remains unclear. This study investigated how the initial fruiting branch rate (IFBR) and initial fruit number per cluster (INFC) regulate endogenous hormones in the aril and leaflets of mature ‘Shixia’ longan. Key findings reveal the aril as the hormonal sink, accumulating auxin (IAA) and abscisic acid (ABA), while leaves retain IAA precursors (TRP) and conjugates. Higher IFBR and INFC increased the demand for IAA in the aril to support expansion but simultaneously elevated ABA levels in leaves. Notably, IFBR exerted a stronger influence than INFC. These hormonal changes were significantly correlated with the fruit shedding rate and soluble solid content. Overall, the endogenous hormone profile was optimized by maintaining a moderate IFBR of approximately 60% and an INFC within the range of 60 to 80, achieving balance between fruit expansion, leaf vitality, and yield. The results provide a hormonal basis for precise crop load management in longan cultivation.

## 1. Introduction

Fruit development is a complex process governed by plant hormones, which orchestrate key transitions from flowering to senescence [1,2,3,4]. Throughout ontogeny, specific hormones fulfill distinct roles. ABA and ethylene are known to regulate processes such as vegetative growth and floral bud differentiation [5,6], whereas auxin (IAA) and zeatin (ZT) contribute to flower bud formation and early fruit cell division and expansion [7,8,9,10]. The significance of hormonal regulation is further highlighted by the fact that exogenous applications of GA_4+7_ and IAA can induce parthenocarpy [11,12,13]. As development proceeds, the dynamic balance among hormones becomes particularly important during ripening, where ABA and ethylene are key drivers of sugar accumulation and senescence [14,15,16]. Hormonal profiles are not static but vary with developmental stage, cultivar, and environmental conditions, thereby profoundly influencing final fruit quality and stress resilience [17,18]. Thus, endogenous hormone dynamics constitute a central regulatory axis in fruit development.

*Dimocarpus longan* Lour., a tropical evergreen tree, exhibits vigorous vegetative growth and strong fruit setting ability. However, its productivity is frequently compromised by alternate bearing. Longan inflorescences form large conical corymbs demanding substantial nutrient allocation, while its pinnate compound leaves exhibit high photosynthetic efficiency. This creates a complex interplay between whole-plant vigor and individual fruiting branch (FB) autonomy, resulting in the coexistence of vigorous and feeble growth between the entire plant and individual FBs. To mitigate high fruit shedding rates (FSRs) and alternate bearing, strategies such as flower/fruit thinning and tree vigor (TVR) management have been prioritized [19,20]. Key parameters like the initial fruiting branch rate (IFBR) and initial fruit number per cluster (INFC) critically determine yield stability and fruit quality [21]. However, the hormonal mechanisms underlying these effects remain poorly understood.

Optimal flower and fruit retention ensure suitable longan yield, improve postharvest storability, and increase commercial value through superior fruit size and weight [22,23]. However, scant research has been conducted on how endogenous hormones modulate FB growth, leaf development, and fruit quality in longan. Early studies identified ABA and auxin ratios as regulators of fruit abscission in young ‘Shuizhang’ longan [24]. The content of cytokinins (CTKs) was greater in the early stages of ‘Chuliang’ longan fruit development. Furthermore, the occurrence of fruit drop in this cultivar might be associated with lower contents of auxin and GAs. Nevertheless, the swift increase in auxin content in the later stages of development may promote rapid fruit expansion [25]. Notably, aril expansion in mature longan fruits replaces kernel and pericarp growth as the primary sink, yet hormone profiles in aril tissues and FB leaves during this critical stage remain uncharacterized. Furthermore, the interplay between hormonal dynamics, FB vigor, fruit setting rate, and fruit quality has not been systematically explored.

In this study, a single-factor randomized block experiment was conducted with three IFBR levels (blocks) and three INFC levels (treatments). A total of nine “block × treatment” combinations were employed to investigate the effects of IFBR and INFC on endogenous hormones, as well as their interconnections, in the mature fruit aril and FB leaflets. This study aimed to explore the relationships between the fruiting load and growth of the FBs and fruit quality, with a focus on endogenous hormones. This study provides a reference for the future development and application of chemical regulation technology for longan fruit development under varying fruiting load conditions.

## 2. Results

To ensure clarity in presenting the results, key terms related to the experimental design are defined here. This study employed three levels of IFBR: low IFBR: 30% (referred to in the results as ‘block 1’); medium IFBR: 60% (‘block 2’); high IFBR: 90% (‘block 3’). Within each IFBR group, three levels of INFC were applied: low INFC: 30–40 (referred to as ‘treatment 1’); medium INFC: 60–80 (‘treatment 2’); and high INFC: 100–120 (‘treatment 3’). The nine unique combinations of the IFBR and INFC levels are labeled H11 to H33 (e.g., H11 denotes low IFBR with low INFC). The detailed design matrix is provided in the Section 4.1.

### 2.1. Comparison of the Growth, Fruit Bearing, and Fruit Quality of FBs Under Different Combinations During Maturity

**Figure 1:**
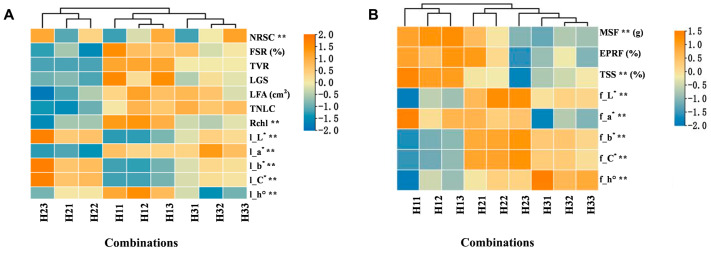
Comparison of TVR, FB leaflet growth, and fruit bearing (**A**) and fruit quality (**B**) of each combination at the mature stage. Note: NRSC, number of remaining fruits in a single cluster; FSR, fruit shedding rate; TVR, tree vigor; LGS, leaflet glossiness; LFA, leaflet area; TNLC, total number of leaflets in a single cluster; Rchl, relative chlorophyll content; l_L*, L value of leaves; l_a*, a* value of leaves; l_b*, b* value of leaves; l_C*, C* value of leaves; l_h°, h° value of leaves; MSF, mass of a single fruit; EPRF, edible portion rate of a single fruit; TSS, total soluble solids; f_L*, L value of fruits; f_a*, a* value of fruits; f_b*, b* value of fruits; f_C*, C* value of fruits; f_h°, h° value of fruits; the ** marked in the upper right corner indicates the highly significant difference between the combinations (*p* < 0.01), the same as below.

Combinations H11, H21, and H31 presented the lowest number of remaining fruits in the single cluster (NRSC), whereas H12, H22, and H32 presented somewhat higher NRSC values. H13, H23, and H33 presented the highest NRSC values (Figure 1A). This is consistent with the difference in the INFC, and the difference among combinations was highly significant (*p* < 0.01). Block 1 presented the highest FSR, followed by block 3, whereas block 2 presented the lowest FSR. The highest values for TVR, FSR, leaflet glossiness (LGS), leaflet area (LFA), and relative chlorophyll content (Rchl) were observed in block 1, followed by block 3, and the lowest values were found in block 2. The total number of leaflets in a single cluster (TNLC) was the lowest in block 2, whereas blocks 1 and 3 presented relatively high and similar TNLC values. The leaves in block 1 had the lowest values for l_L*, l_b*, and l_C* but the highest value for l_h°. Additionally, the color of the leaves was dark green. The leaves in block 2 exhibited a yellowish-green color, with the highest values for l_L*, l_b*, and l_C* and the lowest values for l_a*. Moreover, the leaves in block 3 were grayish-green, with medium values for l_L*, l_b*, and l_C* and the highest value for l_a*.

Cluster analysis revealed that all combinations within blocks 1, 2, and 3 were classified into distinct categories (strong, weak, and moderate, respectively) based on their TVR levels. Among the different treatments within the same block, with the exception of the NRSC, the other leaf growth indices and FSR were essentially consistent with the TVR, and the differences were significant (*p* < 0.01), indicating that differences among the blocks had a significant impact on the FSR and growth of the FB leaflets.

With the exception of the EPRF, there were significant differences in the other fruit quality indicators among the combinations (*p* < 0.01) (Figure 1B). The MSF, EPRF, and total soluble solid content (TSS) of each treatment fruit in block 1 were the highest, followed by those in block 2; those in block 3 were the lowest, showing slow fruit expansion and growth detention. In terms of the chromaticity index, each combination in block 2 had the highest values of f_L*, f_a*, f_b*, and f_C*, exhibiting a bright and rich yellowish-brown color on the fruit surface. In block 1, the values of f_L*, f_b*, f_C*, and f_h° were the lowest, whereas f_a* was relatively high, resulting in a dull greenish-brown color on the fruit surface. In block 3, the values of f_L*, f_b*, and f_C* were all moderate, with the lowest value of f_a* and the highest value of f_h°, resulting in a yellowish-green color on the fruit surface. The cluster analysis results indicate that H11–H13 presented the most superior characteristics in terms of MSF, EPRF, and TSS content, followed by H21 and H22, whereas H23–H33 presented the lowest values in these aspects. The consistency of fruit quality within the same block was high, but H23 in block 2 had lower MSF, EPRF, and TSS contents, which is similar to the combinations in block 3. Block 1 had the lowest fruit setting rate, the most rapid fruit growth, and the earliest maturity. Conversely, block 3 presented the highest fruit setting rate and slowest fruit ripening. However, the TSS content and appearance of the fruits in block 3 did not reach normal maturity levels, leading to sugar reduction and signs of aging.

Among the nine combinations, the NRSC ranged from 23.38 to 79.30, with a high coefficient of variation of 44.91%. Moreover, the FSR ranged from 23.00% to 40.75%, with a coefficient of variation of 18.15% (Table 1). The coefficients of variation for the growth indices of the FB leaflets, including TVR, TNLC, LFA, LGS, Rchl, l_L*, l_a*, l_b*, l_C*, and l_h°, ranged from 3.02% to 28.87%. Among the fruit quality indices, the MSF, EPRF, and TSS content ranged from 5.52 to 7.49 g, 56.46% to 64.94%, and 18.34% to 23.12%, respectively. The coefficient of variation for all fruit quality indices ranged from 2.12% to 11.98%. Different combinations of fruit clusters significantly differed in terms of growth, fruit bearing ability, and fruit quality (Figure 1).

### 2.2. Comparison of the Endogenous Hormone Contents in Fruit Aril and FB Leaflets at the Mature Stage Under Different Combinations

**Figure 2:**
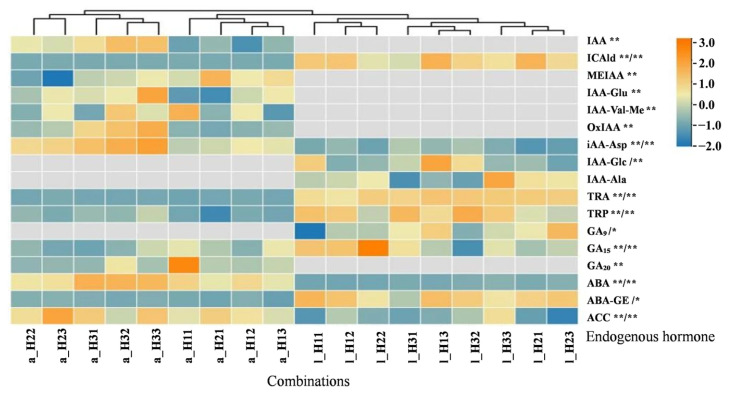
Comparison of the endogenous hormone contents in fruits and FB leaflets of each combination at mature stage. Note: Gray cells in the plot indicate those not detected; the ordinate suffix * or ** and /* or /** indicates significant (*p* < 0.05) or highly significant (*p* < 0.01) differences, respectively, in the fruit and leaf between combinations. a_ and l_ indicate the aril and leaf, respectively, the same as below.

A total of 17 endogenous hormones were detected, with 14 endogenous hormones detected in the fruit and 11 detected in the leaves (Figure 2). The hormones can be classified into four types: (1) Auxins such as Indole-3-acetic acid (IAA), IAA methyl ester (MEIAA), IAA glutamate (IAA-Glu), IAA-valine methyl ester (IAA-Val-Me), and OxIAA were detected only in the fruit aril, whereas IAA-1-glucosyl ester (IAA-Glc) and IAA- L-alanine (IAA-Ala) were detected only in the leaves. Except for IAA-Ala, the other hormones significantly differed among the treatments (*p* < 0.01). Indole-3-carboxaldehyde (ICAld), IAA-L-aspartic acid (IAA-Asp), Tryptamine (TRA), and L-Tryptophan (TRP) were detected in both the aril and leaves, but the content of IAA-Asp was significantly greater in the aril than in the leaves, whereas synthetic precursor substances ICAld, TRA, and TRP presented the opposite trend. The results indicate that, at the mature stage, leaves contained minimal levels of active IAA, while high contents of partially synthesized precursor substances, as well as transformation and degradation products (IAA-Asp, IAA-Glc, IAA-Ala), were found in the leaves. In contrast, the fruits contained relatively high concentrations of active IAA, as well as its transformation and degradation products (IAA-Asp, IAA-Glu, IAA-Val-Me, OxIAA, and MEIAA) [26,27]. (2) Gibberellins: GA_15_ and GA_20_ were present mainly in the aril, whereas GA_9_ and GA_15_ were present mainly in the leaves. In most combinations, the content of GA_15_ in the leaves was greater than that in the aril. (3) Abscisic acids: ABA and ABA-GE were detected in both the leaves and the aril. The ABA content in the aril was greater than in the leaves, whereas the ABA-GE content in the leaves was greater than in the aril. (4) Ethylene: The content of ACC, a direct precursor for ethylene synthesis, was significantly greater in mature fruits than in leaves. The cluster analysis results indicate that combinations H11–H13 and H21 resulted in the lowest IAA content in the fruit, moderate GA content, and relatively low ACC content. Moreover, H22 and H23 presented moderate IAA contents, the lowest GA contents, and the highest ACC contents. Combinations H31–H33 had the highest levels of auxin and ABA and moderate levels of ACC in the fruits. The clustering features of several hormones were not very distinct in the leaves, indicating that the mature fruit was still the growth center of the FBs. Furthermore, the combinations with high levels of IFBR and INFC presented relatively high levels of IAA and ABA.

The coefficient of variation in the endogenous hormone contents in the leaflets of each combination ranged from 8.27% to 57.34%. Among the combinations, GA_15_ had the highest coefficient of variation, whereas IAA-Ala had the lowest coefficient of variation. Additionally, the coefficient of variation in the endogenous hormone content in the aril ranged from 10.33% to 50.53%, with IAA-Val-Me and ABA-GE having the highest and lowest coefficients of variation, respectively (Table 2). (1) The content of IAA-Asp in the fruits (56.30–136.00 ng·g^−1^) was 3–4 times greater than in the leaflets (14.8–48.1 ng·g^−1^). The levels of ICAld and TRA (0.44–0.90 and 0.08–0.40 ng·g^−1^, respectively) were much lower in the fruits than in the leaflets (66.75–145.50 and 4.09–5.85 ng·g^−1^, respectively). Additionally, the level of TRP (1.46 × 10^4^–2.74 × 10^4^ ng·g^−1^) in the fruits was slightly lower than in the leaflets (2.71 × 10^4^–4.35 × 10^4^ ng·g^−1^). These findings indicate that the synthesis of IAA with certain precursor substances (ICAld, TRA, and TRP) in the leaflet may be inhibited, leading to the accumulation of IAA degradation products (IAA-Glc and IAA-Ala). (2) The coefficient of variation in GA_15_ in the aril (57.34%) was greater than in the leaflets (41.90%), whereas the difference in the GA_9_ content in the leaflets and the GA_20_ content in the aril was not significant. (3) The ABA content in the aril, ranging from 487.50 to 944.50 ng·g^−1^, was 6.5–8.2 times greater than in the leaflets, which ranged from 59.65 to 145.50 ng·g^−1^. However, the content of ABA-GE in the aril (127.50–176.50 ng·g^−1^) was lower than in the leaflets (244.50–512.00 ng·g^−1^), indicating that the content of ABA-GE in the aril was only 0.3–0.5 times greater than in the leaflets. (4) The ACC content in the leaflets ranged from 45.80 to 86.40 ng·g^−1^, which was slightly lower than in the aril (74.10–107.50 ng·g^−1^). In summary, the mature longan aril is the center of IAA and ABA accumulation. Additionally, the production of ethylene was greater in the aril than in the leaflets, whereas the difference in GAs was not significant.

### 2.3. Synergistic Effects of FB Growth, Fruiting and Quality Indicators on Endogenous Hormones in the Fruit Aril and FB Leaflets

#### 2.3.1. Regression Analysis of Endogenous Hormones on Related Growth, Fruiting, and Quality

Linear regression analysis was conducted to investigate the relationships among endogenous hormones and plant vigor, FB growth, fruit bearing, and fruit quality. The levels of 22 endogenous hormones were used as dependent variables, and various indicators of plant growth and fruit quality were used as independent variables (Table 3). Independent variables (alternative covariate indicators) that had significant linear regression relationships with endogenous hormones were obtained. The regression effects of independent variables X_1_, X_2_ … X_n_ on hormone indicators decreased sequentially, while the regression relationships were all significant (*p* < 0.05). The *R*^2^ values of all regression models ranged from 0.469 to 0.990, indicating that the explanatory power of the synergistic effect of each variable in each model on the total variation in the dependent variable between combinations was 46.9% to 99.0% (*R*^2^ value × 100%). The higher the explanatory power, the greater the impact of other confounding factors beyond the treatment and group on the total variation between combinations. In addition, the *p* value range of all regression models was 0–0.042, indicating that each model was statistically significant.

The regression results indicate that (1) l_h° was first correlated with the a_IAA content, the ratio of a_IAA to a_ACC (a_IAA/ACC) and l_GAs/ABA and second correlated with a_ACC, resulting in a greenish color on the FB leaflets, with relatively low levels of a_IAA and a_IAA/ACC, and relatively high levels of ACC and l_GAs/ABA. (2) f_a* was first correlated with a_IAA-Glu, a_OxIAA, a_IAA-Asp, a_TRP, a_ABA, and l_ACC and second correlated with a_MEIAA, resulting in a brownish color on the fruit surface, with low levels of a_IAA synthesis precursors and conversion substances, as well as l_ACC and a_ABA. (3) LGS was first correlated with a_GA_15_ and l_IAA-Glc, second correlated with a_ABA/ACC, and third correlated with l_ABA/ACC, indicating that the higher the LGS, the lower the content of a_ABA/ACC, and the higher the contents of a_GA_15_, l_IAA-Glc and l_ABA/ACC. (4) MSF was first correlated with l_ICAld and l_ABA and second correlated with l_ACC, indicating that, the higher the MSF, the higher the content of l_ICAld and l_ACC, and the lower the content of l_ABA. Additionally, when the value of l_a* was high (the leaflets tended to look grayish-green in color), a_ABA/ACC and l_TRP increased, whereas the ratio of l_GAs/ACC decreased.

In addition, the NRSC was positively correlated with a_IAA-Glu and l_TRP. Fruits with higher EPRF values contained higher content of a_MEIAA and lower content of a_IAA-Asp. Fruits with relatively high TSS contents presented relatively low a_ACC and l_ABA/ACC values. The FSR was highly significantly negatively correlated with a_IAA/ABA (*p* = 0.009), whereas the TNLC was positively correlated with l_IAA-Asp. In summary, hormone indicators are closely related to the growth and fruiting status of the FB, as well as the internal and external qualities of the fruit.

#### 2.3.2. Correlations Between Endogenous Hormones, Plant Growth, Fruiting, and Fruit Quality

Bivariate correlation analysis revealed that (1) a_IAA was significantly positively correlated with l_ABA and a_ABA (*r* = 0.711*, 0.673*), and highly significant negative correlations existed between MSF and a_IAA and l_ABA (*r* = −0.896**, −0.839**). Additionally, a highly significant positive correlation existed between f_h° and a_IAA (*r* = 0.865**). These findings indicate that fruit growth and leaf senescence mutually promote each other and that fruit growth and senescence also develop synchronously at the mature stage. Furthermore, the greater the a_IAA, the stronger the green retention ability of the fruit pericarp. (2) There was a highly significant positive correlation (*r* = 0.987**) between the NRSC and FBRT. Additionally, the stronger the TVR, the greater the FSR (*r* = 0.899**), but the relationships between the FSR and blocks and treatments were not significant (*r* = −0.339, −0.312). (3) The correlation coefficients between the TVR and l_L*, l_a*, l_b*, l_C*, and LGS were −0.913**, 0.751*, −0.933**, −0.941**, and 0.897**, respectively, indicating that the stronger the TVR, the darker the color of the FB leaflets. (4) The Rchl content in the leaves was significantly positively correlated with the TVR (r = 0.924**), l_h° (*r* = 0.816**), TSS content (r = 0.931**), and MSF (r = 0.777*). (5) a_IAA/ABA was significantly positively correlated with a_L*, a_b*, and a_C*, with correlation coefficients of 0.699*, 0.698* and 0.715*, respectively, indicating that when the a_IAA/ABA content increased, the pericarp developed a yellow color. In summary, hormones are significantly related to the growth and fruiting of FBs and fruit quality.

### 2.4. Results of Covariance Main Effects Test with Endogenous Hormone Indicators as the Dependent Variable

Univariate covariance analysis was conducted to determine the hormone indicators that were significantly affected by the block (IFBR), treatment (INFC), or covariate (Table 4). Among the 22 hormones in Table 3, only 9 were significantly affected by the block and treatment: a_IAA, a_ABA, a_ABA/ACC, a_IAA/ABA, a_IAA-Asp, a_IAA-Glu, a_OxIAA, l_ABA, and l_TRP. Specifically, a_IAA/ABA was significantly affected by the FSR (*p* = 0.008), and a_GA_20_ was significantly affected by f_L* (*p* = 0.043) but was not significantly related to the block or treatment. In addition, for other hormone indicators whose regression models were not produced, the main effects test results of the analysis of variance were not significant. Except for a_IAA/ABA and a_GA_20_, the differences in the other eight hormones among the treatments were not affected by the growth and fruiting of branches or fruit quality, and were affected mainly by the blocks or treatments.

The nine hormones with significant main effect test results were all significantly affected by the block (IFBR) (*p* = 0–0.025). Among these, four hormones (a_IAA-Asp, a_IAA-Glu, a_IAA/ABA, l_ABA) were also significantly affected by the treatment (INFC) (*p* = 0.007–0.036). This indicates a greater difference in the endogenous hormone levels among the different IFBRs compared to the INFC. Among the nine endogenous hormones, six were detected in the aril. Covariance analysis (Table 4) further confirmed that the variation in these hormones was more significantly affected by the fruit load treatments (IFBR and INFC) in the aril than in the leaflet. In addition, the *R*^2^ value of the main effect test for the above endogenous hormone indicators ranged from 0.888 to 0.982, indicating that the combined effect of fixed variables (block + treatment) and covariates contributed 88.8% to 98.2% of the total variation among the combinations (*R*^2^ value × 100%).

### 2.5. Comparison of Estimated Marginal Means of Endogenous Hormone Indicators in Leaflets and Aril Significantly Affected by the Block and Treatment

The estimated marginal means (*p* < 0.05) of hormone indicators significantly affected by the block and treatment after excluding covariate interference are shown in Table 5. Combined with the regression and correlation analyses in Section 2.3.1, the results revealed the following:

Among the blocks, the levels of a_IAA, a_IAA-Asp, a_OxIAA, and l_ABA were positively correlated with increasing IFBR. However, hormone levels in block 3 (90% IFBR) were significantly higher than those in both block 1 (30% IFBR) and block 2 (60% IFBR). With increasing IFBR, the levels of a_IAA and l_ABA increased, and the metabolism of a_IAA became more vigorous. In other words, the lower the MSF, the weaker the fruit expansion ability, and the greater the requirement for IAA. Additionally, the degradation products of a_IAA also significantly increased, whereas leaf senescence was more obvious. Furthermore, the trends in the changes in the levels of a_ABA, a_ABA/ACC, a_IAA-Glu, and l_TRP among the three blocks were relatively consistent. The levels of hormones in block 3 were the highest, whereas those in block 2 were the lowest. These findings indicate that the a_ABA content required for fruit ripening was the highest in block 3, and the degradation product of IAA, a_IAA-Glu, significantly increased in the fruit aril. The precursor of IAA, l_TRP, significantly accumulated in the leaves, whereas IAA was not detected in the three blocks (Figure 2), indicating that IAA biosynthesis was blocked. In addition, a_IAA/ABA was lowest in block 2 and highest in block 1, indicating that, under the same FSR (covariation), the color of the pericarp in block 2 tended to be dim yellow, and that in block 1 was light yellow (Section 2.3.2). In summary, the change in the auxin content in the aril is consistent with the pattern observed at the block level among different blocks. As the fruit growth rate decreases, the level of IAA required increases. The a_ABA level was lowest in block 2 (with a reasonably moderate ripening process), moderate in block 1, and highest in block 3. This suggests that, when the ripening process is too fast or too slow, the level of a_ABA increases.

Among the treatments, except for a_IAA/ABA, a_IAA-ASP, a_IAA-Glu, and l_ABA, there was no significant difference in the other endogenous hormones among the treatments (Table 5). Under the condition of a consistent FSR (covariate), treatment 2 had the lowest level of a_IAA/ABA, with a light-yellow pericarp. Moreover, treatment 1 had the highest level of a_IAA/ABA, with a dark yellow pericarp. The levels of a_IAA-ASP, a_IAA-Glu, and l_ABA were relatively low and similar between treatments 1 and 2. This was accompanied by a brownish pericarp; lower NRSC, FSR, and TNLC; and higher MSF and EPRF. However, there was a significant increase in the levels of the above hormones in treatment 3. There was no significant difference in other hormones among the treatments, among which auxins increased with increasing treatment levels (INFC).

## 3. Discussion

### 3.1. The Fruiting Load Affects the Growth and Fruit Quality of Longan FBs in the Mature Stage

Alternate bearing poses a significant challenge in longan cultivation. Flower and fruit thinning during the reproductive phases remains the primary strategy employed to regulate plant load, mitigate growth–fruit competition, and enhance longan fruit quality. The IFBR and INFC are two important technical parameters for thinning flowers and fruits. Xu et al. identified optimal thresholds of 32% IFBR and 60–70% INFC for ‘Lidongben’ longan [28]. Liu demonstrated that flower thinning improves the female flower ratio, fruit setting rate, branch nutrient status, and ultimate fruit quality [29]. Han et al. further refined these parameters, proposing a synergistic balance of 50–60% IFBR and the approximately 60% INFC to optimize plant load–fruit quality equilibrium [21]. However, the above studies did not investigate the impact of fruit load on the growth of leaves in FBs or the impact of differential INFC treatments on the FSR. The negative correlation between IFBR and key traits (MSF, TVR) fundamentally stems from source limitation. As the number of fruiting branches increases, photoassimilates are partitioned among more sinks, reducing the resource share per fruit and compromising overall TVR. The latter is directly observable as leaf chlorosis or dullness, indicating assimilate diversion away from leaf maintenance. The dominant effect of IFBR over INFC reveals that the whole-plant sink strength primarily dictates the physiological state, with INFC playing a secondary modulating role. At the cluster level, high INFC intensifies intra-plant competition, leading to poorer fruit quality (lower MSF, EPRF, and TSS). The associated brighter pericarp coloration likely reflects a precocious or stress-related pigmentation shift rather than superior maturity. Conversely, high leaf chlorophyll content signifies robust source strength, which is essential for achieving higher fruit mass and sugar accumulation.

### 3.2. Endogenous Hormones Affect the Growth of FBs and Fruit Quality

Endogenous hormones are central regulators of fruit development and quality. While their roles are well-established in many species [30,31,32,33,34], the hormonal interplay within longan FBs during maturation, particularly in response to fruiting load, remains unclear. Our study fills this gap by revealing a distinct hormonal division of labor: FB leaflets contained no detectable IAA but high levels of its precursor TRP, whereas the aril accumulated high concentrations of IAA, ABA, and ACC. This pattern establishes the aril as the dominant hormone sink and growth center during maturation. Zhou et al. established correlations between endogenous hormone levels and developmental stages in longan fruit [25], but this study did not investigate the relationships between endogenous hormones and the growth of FBs, fruiting characteristics, or fruit quality during the maturation stage. It is generally believed that ABA promotes sugar accumulation and ripening at the early stage of fruit ripening, whereas ETH promotes fruit senescence at the later stage of fruit ripening [35,36,37]. Our findings reveal that FB leaflets did not contain IAA but contained a greater level of the synthetic precursor substance TRP, whereas aril showed relatively high content of IAA and its conversion products. Both the leaves and aril contained GAs, ABA, and ACC, but the contents of ABA and ACC in the aril were greater than those in the leaves, indicating that the growth and development center of the FB is in the aril and that the sugar synthesized by the leaves is transferred to and accumulates in the aril.

Our correlation analyses substantiate this model and link hormones to concrete quality traits. The ratio of a_IAA/ABA was negatively correlated with FSR, aligning with prior findings [24], while a_ACC and l_ABA/ACC were closely tied to TSS accumulation. Importantly, l_ABA levels negatively affected MSF. These hormonal shifts were visibly manifested: a favorable IAA/ABA balance correlated with the bright yellowish-brown pericarp color characteristic of optimal ripening, whereas elevated a_ABA/ACC and l_ABA were linked to leaf yellowing (higher b* value) and diminished gloss, phenotypic hallmarks of senescence. Thus, a_IAA appears to coordinate with l_ABA and a_ABA to mutually drive aril growth and leaf senescence, with colorimetric data providing the direct phenotypic validation of the underlying hormonal states. In summary, during the maturation stages, when a_IAA, a_ABA, a_IAA/ABA, a_ABA/ACC, l_ABA, and l_ABA/ACC are low, the fruits are relatively larger and ripen earlier, and the FSR and TSS contents are greater. Moreover, longan fruits appear greenish brown in color (green during the young fruit period), showing rapid development and strong fruit expansion potential; the TVR is strong; and the leaves are dark green or strongly green.

### 3.3. The Fruiting Load Affects Endogenous Hormones in FB Leaflets and the Aril

The variation in fruiting load (IFBR and INFC) observed in this study was found to exert a profound influence on the endogenous hormone profiles in both the aril and leaflets, which can be interpreted through the lens of source–sink relationships and assimilate competition. Our results demonstrate that a high fruiting load (90% IFBR) led to significant accumulations of IAA and its degradation products (e.g., OxIAA, IAA-Asp) in the aril, concomitant with an increase in ABA. This suggests a state of “assimilate competition-induced stress.” Under high sink demand, the rapid expansion of a large number of fruits may require sustained high levels of IAA to mobilize photosynthates and maintain cell elongation. However, the simultaneous surge in ABA and IAA catabolites could indicate a hormonal shift towards ripening and senescence, potentially triggered by resource limitation [38]. This paradoxical increase in both growth-promoting (IAA) and senescence-promoting (ABA) hormones may explain the observed “growth detention” in high-load conditions, where fruit development is slow despite high hormonal levels, as the tree struggles to allocate limited resources across excessive sinks. This response contrasts with mechanisms reported in grapes, where fruit removal elevates leaf CTKs and reduces GAs [39], highlighting species-specific hormonal strategies in managing source–sink competition.

Furthermore, the absence of detectable IAA in FB leaflets, despite the accumulation of its precursor TRP, suggests a possible inhibition of IAA biosynthesis or, more likely, a rapid and efficient translocation of synthesized IAA from leaves (source) to the dominant sink organs (fruits). This source–sink dynamic ensures that hormonal resources are prioritized for fruit development, potentially at the cost of leaf maintenance, which aligns with the observed decline in leaf vitality under high loads. This interpretation is further supported by the concurrent accumulation of IAA conjugates (IAA-Asp and IAA-Glc) in leaves, which indicates active IAA catabolism or deactivation pathways likely contributing to the low free IAA pool. While methodological limitations in detection cannot be entirely ruled out, the high sensitivity of our LC-MS/MS analysis, which successfully quantified IAA in the aril and numerous other hormones in leaves, makes this explanation less probable. The consistent pattern across all samples strongly suggests a genuine biological regulation rather than an analytical artifact.

Many studies have been conducted on the content and changes in endogenous hormones during fruit development, but few have reported on the content and interrelationships of endogenous hormones in fruits and leaves, or on the effects of load differences on endogenous hormones in plant leaves and fruits. Research on tomatoes suggests that the content of ABA in leaves has no significant effect on fruit shedding [40]. Bangerth suggested that the self-starting and self-inhibiting components of IAA can replace ethylene as the primary driving force for the initiation of young fruit abscission [41]. The fruit load of grape significantly affects the levels of CTKs and GAs in the leaves. The removal of fruit from potted cuttings of *Vitis vinifera* L. increased the concentration of CTKs in the leaf and decreased the amount of GA-like substances [39]. In this study, compared with the INFC, the effect of the IFBR on plant leaf growth and fruit quality was more significant and extensive. Based on the regression relationships, correlations, and main effect analysis, our findings illuminate the hormonal mechanisms underlying optimal and suboptimal fruit loads in longan: (1) The hormonal signature of an optimal load (60% IFBR) appears to be one of balance and efficiency. The lowest levels of a_ABA, a_IAA/ABA, a_ABA/ACC, a_IAA-Glu, and l_TRP, coupled with moderate a_IAA and l_ABA, suggest a state where fruit expansion proceeds with minimal stress- and senescence-related hormonal induction. This balanced profile likely supports normal pericarp cell renewal and the development of the characteristic brown coloration, while allowing the fruiting branch (FB) leaflets to retain chlorophyll and maintain photosynthetic function. In contrast, a low load (30% IFBR), while yielding the highest MSF, may represent an underutilized sink capacity in which hormonal drivers for expansion (a_IAA) and maturation (a_ABA) are minimal but not optimally coordinated for quality development. Conversely, the high-load (90% IFBR) scenario triggers a pronounced hormonal stress response: the significant increases in both a_IAA and a_ABA indicate a physiological conflict where the tree attempts to support excessive sink demand (via elevated IAA) while simultaneously activating ABA-mediated pathways associated with resource limitation and senescence. This imbalance manifests as growth detention, darkened, aging-prone leaves, and compromised fruit quality. (2) The influence of INFC within a given IFBR further refines this picture. Treatment 2 (60-80 INFC), which aligned with the best overall fruit quality, exhibited the lowest a_IAA/ABA ratio, promoting a pericarp color shift towards maturity. The low l_ABA in both treatments 1 and 2 suggests that maintaining leaf vitality is achievable across a range of INFCs, provided the whole-plant load (IFBR) is not excessive. The marked increase in a_IAA-Asp, a_IAA-Glu, and l_ABA in treatment 3 (high INFC) indicates that excessive cluster-level density exacerbates IAA catabolism and leaf ABA accumulation, even under a moderate whole-plant load, leading to the poorer quality outcomes observed.

Notably, the a_IAA/ABA ratio was strongly negatively correlated with FSR. Statistical modeling confirmed that this relationship was significant even after accounting for the effects of blocks and treatments, highlighting its central role in determining fruit retention.

### 3.4. Suggestions for Field Management in Longan Fruiting Stages

Based on our integrated analysis, maintaining a balance between source (leaf) and sink (fruit) is paramount for ‘Shixia’ longan. We recommend that flower and fruit thinning prioritize achieving an IFBR of approximately 60%, followed by adjusting the INFC to 60–80 fruits per cluster, based on individual fruiting branch vigor. This strategy, validated under our experimental conditions in Guangdong, China, optimally supports fruit expansion, preserves leaf vitality, and ensures desirable yield and quality. Growers in other contexts should treat these values as a benchmark, adapting them to local conditions.

Beyond immediate management, our study elucidates the endogenous hormonal basis of high-load stress, revealing specific physiological targets for future intervention. The concurrent demand for IAA and accumulation of ABA/ACC under high load provides a strong mechanistic rationale for exploring targeted chemical regulation. Future research should therefore focus on developing cost-effective chemical thinning agents to achieve optimal load and on testing hormone-based strategies—such as IAA-class substances during fruit expansion or modulators of ABA/ethylene action during maturation—to mitigate high-load stress. These directions, grounded in the mechanistic understanding established here, represent promising avenues for translating physiological insights into improved orchard practice.

## 4. Materials and Methods

### 4.1. Plant Materials

The experiment was conducted in a standard orchard of the Fruit Tree Research Institute of Guangdong Academy of Agricultural Sciences. A total of nine 15-year-old longan trees (*Dimocarpus longan* cv. ‘Shixia’) were selected. These trees were of moderate and comparable vigor, grown on a gentle slope of approximately 10°, with a crown size of 5–6 m × 5–6 m, plant height of 2–3 m, flowering rate of 75% to 100%, and initial fruit setting rate of 70% to 100%.

A single-factor randomized block design was adopted, using IFBR as the block and INFC as the treatment. Three blocks with low (30%), medium (60%), and high (90%) IFBRs and three treatments with low (30–40, averaging 37), medium (60–80, averaging 75), and high (100–120, averaging 116) INFC were designed. Each block contained three replicate trees, resulting in a total of nine experimental trees (3 blocks × 3 trees per block). For each block, 3 treatment levels were arranged, resulting in a total of 9 combinations of “block × treatment”, labeled H11–H33 (Table 6). For each combination within each tree, 3 fruit clusters were labeled and managed accordingly, yielding 9 observational units per tree (3 INFC treatments × 3 clusters) and 81 observational units for the entire experiment (9 trees × 9 clusters).

### 4.2. Measurement of Growth and Fruiting of FBs

The length and width of the leaflets, TNLC, and NRSC were measured by counting or measuring each FB for each combination (H11–H33).

The leaf area was calculated according to the following formula:

Leaf area (LFA) = π × 1/2 leaflet length × 1/2 width of leaflet

The FSR was calculated according to the following formula:

FSR = (INFC − NRSC)/INFC × 100%.

Rchl: A composite sample was prepared for each FB. This sample consisted of six leaflets (specifically, the second and third leaflets from the second and third pairs of compound leaves on the FB). The Rchl of this composite sample was measured as one value per cluster using a PhotosynQ MultispeQ multifunctional plant-measuring instrument (East Lansing, MI, USA). Thus, 81 Rchl values were obtained, corresponding to the 81 experimental clusters. The same sampling procedure was used for leaf material collection in the subsequent endogenous hormone analysis.

Chromaticity: A 3nh spectrophotometer (Shenzhen, China) was used to measure the leaf chromaticity values (l_L*, l_a*, l_b*, l_C*, l_h°). L* represents brightness (0–50–100, black–gray–white); a*, the difference between red and green (−100–0–+100, green–gray–red); b*, the difference between yellow and blue (−100–0–+100, blue–gray–yellow); C*, the saturation, which is the degree of intensity (0–100%, light–dense); and h°, the color tone angle (0~360°, 0° purple~90° yellow~180° green~270° blue). Also, the average of the top, middle, and bottom points on the front of each leaf was taken. The above indicators were based on a single cluster as the value unit, with 9 repeated clusters in each combination (block × treatment).

### 4.3. Measurements of Tree Vigor and IFBR

LGS and TVR when ripening: Qualitative description and quantitative assignment methods were used to measure the LGS and TVR. LGS: 1—nonglossy; 2—slightly glossy; 3—glossy. TVR: 1—declined tree, with yellow or yellowish–green leaves, small, nonglossy, and thin leaf canopy; 2—weak tree, with yellowish–green or nonglossy green leaves, small, and thin leaf canopy; 3—moderately weak TVR, with nonglossy green leaves, small leaves, and moderate leaf canopy thickness; 4—medium TVR, green leaves with slight luster, medium size, and moderate leaf canopy thickness; 5—medium to strong TVR, with dark green leaves, glossy or slightly glossy, larger, and thicker leaf canopy. The TVR and LGS were assessed at the mature fruit stage as indicators of the trees‘ overall physiological response to the imposed fruiting loads.

IFBR: On the basis of production experience, 15–18 effective FBs per cubic meter were taken as the base [42], and the IFBR was calculated as the ratio (%) of the actual number of FBs to the base, for example, 40%: 6–7 fruit clusters per square meter; 50%: 8–9 fruit clusters per square meter; 60%: 9–10 fruit clusters per square meter; and so on, where 100% is the total fruiting rate.

The TVR and individual fruiting rates of plants were measured in blocks, with no duplicates among plants. However, there are 3 identical TVR replicates among the combinations in the same block. Leaf glossiness was measured in units of a single cluster, with 9 repeated clusters per combination.

### 4.4. Measurement of Fruit Quality

For each combination (H11–H33), nine fruit clusters (replicates) were randomly selected. From each cluster, eight fruits were further randomly sampled for quality measurements. The average of the eight fruits was taken as the value for that individual cluster. Consequently, for each combination, nine cluster-level values (each being the mean of eight fruits) were obtained for subsequent analysis. The mass of a single fruit (MSF), fruit chromaticity values (f_L*, f_a*, f_b*, f_C*, f_h°) (mean of three measurement points on the equatorial plane of the fruit), and edible portion rate in a single fruit (EPRF) = (MSF − pericarp weight − seed weight)/MSF × 100% were determined. The TSS of the aril was measured from the juice using a digital refractometer (ATAGO-32 α; Tokyo, Japan).

### 4.5. Detection of Endogenous Hormones in the Leaves and Aril

Sampling: (1) Leaves: The second and third leaflets of the second and third pairs of leaves were collected from each combination (H11–H33) with 10 cluster repeats during the mature period. The leaflets were mixed and frozen immediately in liquid N_2_ after being washed with Milli-Q water and dried with absorbent paper. (2) Aril: One-fourth of the 30 fruits that were randomly selected in each combination were collected and frozen in liquid N_2_. Samples taken from the leaflets and aril were stored at −70 °C, and each combination was repeated three times.

The contents of the hormones in the leaflets and aril were detected using an AB Sciex QTRAP^®^ 6500^+^ LC–MS/MS system (Framingham, MA, USA) [43,44,45]. A Waters ACQUITY UPLC HSS T3 C18 column (1.8 μm, 100 × 2.1 mm^2^) operating at 40 °C was used for separation. The elution program was as follows: solvent A [0.04% (*v*/*v*) acetic acid (Merck, Darmstadt, Germany) in water] and solvent B [0.04% (*v*/*v*) acetic acid in acetonitrile (Merck, Darmstadt, Germany)]; flow rate, 0.35 mL min^−1^; gradient, 0–1 min, 5% B; 1–8 min, 5–95% B; 8–9 min, 95% B; 9–9.1 min, 95–5% B; 9.1–12 min, 5% B. The spectrometer was equipped with an electrospray ionization (ESI) Turbo V ion spray source that operated in both positive and negative modes. Multiple reaction monitoring (MRM) was used to quantify the hormones, where both a quantification and qualitative (confirmation) transition were monitored for each hormone. The positive and negative ion spray voltages were set to 5500 V and −4500 V, respectively, with the source temperature maintained at 550 °C. The method included a curtain gas pressure of 35 psi. Each ion pair was scanned and detected on the basis of the optimized clustering potential (DP) and collision energy (CE).

Method validation was performed to ensure data reliability. The calibration curves for all target hormones showed good linearity (*R*^2^ > 0.99) within the quantification range. The limits of detection (LOD) and quantification (LOQ) were determined for each hormone, confirming the sensitivity of the method. Recovery rates for the hormones in both the leaf and aril fell within an acceptable range (92–108%), and the precision, expressed as the relative standard deviation (RSD), met the requirements for reproducible analysis.

### 4.6. Statistical Analysis

All data in this study were statistically analyzed using Statistical Package for Social Sciences (SPSS), version 23.0 (IBM Corp., Armonk, NY, USA). Assumptions of normality and homogeneity of variances for parametric tests were verified using Shapiro–Wilk and Levene’s tests, respectively. Where necessary, data were transformed to meet these assumptions.

Covariance analysis (ANCOVA) was employed to control for the effects of covariates (e.g., fruit shedding rate, single fruit mass) that were significantly correlated with hormone levels, thereby isolating and more accurately estimating the main effects of IFBR and INFC.

(1) Indicators such as the average value (AV), maximum value (MAX), minimum value (MIN), and coefficient of variation (CV%) were obtained through descriptive statistics. (2) Multiple comparisons between combinations H11–H33 were performed using Duncan’s new multiple range test, and the between indicators were analyzed via Pearson’s correlation. (3) Multiple stepwise regression analysis was conducted to examine the relationships between several endogenous hormones (dependent variables) and the growth and quality indicators of FBs (independent variables). The independent variables that affect hormone indicators were obtained from the regression model as alternative covariates, and variance analysis was conducted on the hormone indicators in the blocks and treatments. (4) Covariance analysis was performed on the hormone indicators via double factor variance analysis in general linear models to exclude uncontrollable factors (such as branch and leaf growth, fruiting traits, and fruit quality) outside of blocks and treatments that may have affected the results.

In this study, heatmap data were standardized prior to analysis, and heatmap visualizations were generated using TBtools-II software, version 2.202.

## 5. Conclusions

This study establishes fruiting load as a critical determinant of longan (cv. ‘Shixia’) growth and quality parameters, with IFBR emerging as the dominant regulatory factor. For trees exhibiting reasonably strong TVR, 60% IFBR and 60–80 INFC are reasonable for achieving optimal balance between plant growth, fruiting, and quality. During the mature stage, the growth center of the FB of longan is located in the fruit aril, and no l_IAA was detected in the FB leaflets. The contents of a_IAA and a_ABA in the aril were much greater than those in the leaves, and the content of a_IAA was significantly positively correlated with that of l_ABA. When the IFBR is high, the content of a_IAA is also high, fruit growth is affected, and late ripening occurs, resulting in a state of growth retention; moreover, the content of l_ABA is also high, TVR is decreased, and yellow leaves occur, leading to low MSF and TSS content in the aril. INFC has a relatively small effect on endogenous hormones in leaves and fruits, mainly affecting l_ABA and a_IAA/ABA. In addition, FBs with 60% IFBR had the lowest contents of a_ABA, a_IAA/ABA, and a_ABA/ACC, whereas FBs with 60–80 INFC had the lowest l_ABA. This indicates that moderate levels of IFBR and INFC are beneficial for meeting the endogenous hormone requirements for fruit expansion, maintaining leaf vigor, and ensuring reasonable yield.

## Figures and Tables

**Table 1:** Descriptive statistics for FB leaflet growth, fruit bearing, and quality indicators of treated clusters from all combinations at the mature stage.

	MIN	MAX	AV	CV (%)		MIN	MAX	AV	CV (%)
NRSC	23.38	79.30	51.64	44.91	l_C*	12.07	22.69	16.34	21.85
FSR/%	23.00	40.75	31.98	18.15	l_h°	106.86	116.89	111.77	3.02
TVR	2.00	4.00	3.00	28.87	MSF/g	5.52	7.49	6.50	11.98
TNLC	66.50	115.40	96.12	20.41	EPRF/%	56.46	64.94	61.42	5.08
LFA/cm^2^	23.47	34.93	30.43	12.54	TSS/%	18.34	23.12	21.02	7.18
LGS	1.30	2.13	1.64	18.40	f_L*	50.60	55.31	53.45	2.83
Rchl	44.81	61.25	53.47	10.46	f_a*	6.22	8.65	7.56	9.90
l_L*	33.36	40.67	36.43	6.63	f_b*	30.70	35.71	33.66	5.39
l_a*	−6.85	−4.51	−5.64	15.25	f_C*	31.97	36.65	34.59	4.99
l_b*	10.79	21.51	15.19	23.51	f_h°	74.21	79.74	77.29	2.12

Note: NRSC, number of remaining fruits in a single cluster; FSR, fruit shedding rate; TVR, tree vigor; LGS, leaflet glossiness; LFA, leaflet area; TNLC, total number of leaflets in a single cluster; Rchl, relative chlorophyll content; l_L*, L value of leaves; l_a*, a* value of leaves; l_b*, b* value of leaves; l_C*, C* value of leaves; l_h°, h° value of leaves; MSF, mass of a single fruit; EPRF, edible portion rate of a single fruit; TSS, total soluble solids; f_L*, L value of fruits; f_a*, a* value of fruits; f_b*, b* value of fruits; f_C*, C* value of fruits; f_h°, h° value of fruits, the same as below.

**Table 2:** Descriptive statistics for the endogenous hormones in FB-leaflet and fruit of each combination at the mature stage.

	In Leaflets				In Fruits			
	MIN	MAX	AV	CV/%	MIN	MAX	AV	CV/%
IAA	-	-	-	-	0.39	0.82	0.61	25.26
IAA-Glu	-	-	-	-	27.00	47.50	36.13	16.66
IAA-Val-Me	-	-	-	-	0.00	0.01	0.01	50.53
OxIAA	-	-	-	-	38.35	134.00	72.19	49.88
IAA-Asp	14.80	52.00	33.37	37.45	56.30	136.00	91.98	29.85
ICAld	57.85	145.50	104.18	29.31	0.44	0.90	0.66	23.88
MEIAA	-	-	-	-	0.07	0.18	0.13	24.00
IAA-Glc	118.50	340.50	197.11	38.22	-	-	-	-
IAA-Ala	6.37	8.46	7.28	8.72	-	-	-	-
TRA	4.09	5.86	5.22	10.77	0.08	0.40	0.23	39.97
TRP (×10^4^)	2.71	4.36	3.54	17.16	1.46	2.74	2.12	16.43
GA_9_	1.37	3.08	2.34	21.51	-	-	-	-
GA_15_	0.01	0.29	0.14	57.34	0.04	0.13	0.08	41.90
GA_20_	-	-	-	-	1.64	3.33	2.00	26.81
ABA	59.65	145.50	95.22	31.68	487.50	944.50	697.11	25.79
ABA-GE	244.50	512.00	418.72	19.63	127.50	176.50	156.94	10.33
ACC	45.80	86.40	61.60	19.48	74.10	107.50	87.76	11.98

Note: IAA-Glu, IAA glutamate; IAA-Val-Me, IAA-valine methyl ester; IAA-Asp, IAA-L-aspartic acid; ICAld, indole-3-carboxaldehyde; MEIAA, IAA methyl ester; IAA-Glc, IAA-1-glucosyl ester; IAA-Ala, IAA- L-alanine; ABA-GE, ABA glucose ester, the same as below. The hyphen (-) indicates that the compound was not detected.

**Table 3:** Identification of growth, fruiting, and quality indicators as covariates for endogenous hormones using stepwise multiple regression.

Dependent Variable (Y)	Independent Variable (X_n_, Alternative Covariate)	Model *R*^2^	Model *P*	Regression Model
a_IAA	l_h°(X_1_)	0.830	0.001	Y = 5.284 − 0.042X_1_
a_IAA-Glu	f_a*(X_1_), NRFC(X_2_), FSR(X_3_)	0.972	0	Y = 54.216 − 4.982X_1_ + 0.174X_2_ + 0.331X_3_
a_MEIAA	EPRF(X_1_), f_a*(X_2_)	0.748	0.016	Y = −0.337 + 0.011X_1_ − 0.03X_2_
a_OxIAA	f_a*(X_1_)	0.842	0	Y = 406.382 − 44.221X_1_
a_IAA-Asp	f_a*(X_1_), EPRF(X_2_)	0.971	0	Y = 478.195 − 27.178X_1_ − 2.944X_2_
a_TRP	f_a*(X_1_)	0.608	0.013	Y = 48,597.86 − 3 628.66X_1_
a_GA_15_	LGS(X_1_)	0.519	0.028	Y = −0.054 + 0.084X_1_
a_GA_20_	f_L*(X_1_), TVR(X_2_)	0.814	0.006	Y = 39.521 − 0.661X_1_ − 0.738X_2_
a_ABA	f_a*(X_1_)	0.573	0.018	Y = 2071.586 − 181.893X_1_
a_ACC	TSS(X_1_), l_h°(X_2_), f_L*(X_3_)	0.968	0	Y = 173.884 − 13.372X_1_ + 3.029X_2_ − 2.687X_3_
a_IAA/ABA	FSR(X_1_)	0.650	0.009	Y = 0.002 − 2.77 × 10^−5^X_1_
a_IAA/ACC	l_h°(X_1_), TSS(X_2_)	0.870	0.002	Y = 0.08 − 0.001X_1_ + 0.001X_2_
a_ABA/ACC	l_a*(X_1_), LGS(X_2_)	0.876	0.002	Y = 34.249 + 3.321X_1_ − 4.535X_2_
l_IAA-Asp	TNLC(X_1_)	0.780	0.002	Y = −20.762 + 0.563X_1_
l_ICAld	MSF(X_1_)	0.475	0.040	Y = −71.398 + 27.007X_1_
l_TRP	l_a*(X_1_), NRFC(X_2_), f_L*(X_3_)	0.990	0	Y = 36,214.141 + 7592.985X_1_ − 76.37X_2_ + 860.979X_3_
l_IAA-Glc	LGS(X_1_)	0.707	0.005	Y = −147.286 + 209.431X_1_
l_ABA	MSF(X_1_)	0.704	0.005	Y = 307.776 − 32.694X_1_
l_ACC	f_a*(X_1_), MSF(X_2_)	0.818	0.006	Y = 146.278 − 20.238X_1_ + 10.497X_2_
l_ABA/ACC	TSS(X_1_), f_h°(X_2_), LGS(X_3_)	0.952	0.001	Y = 28.099 − 0.617X_1_ − 0.193X_2_ + 0.854X_3_
l_GAs/ABA	l_h°(X_1_), f_L*(X_2_), TVR(X_3_)	0.952	0.001	Y = −0.678 + 0.003X_1_ + 0.007X_2_ + 0.008X_3_
l_GAs/ACC	l_ a*(X_1_)	0.469	0.042	Y = −0.018 − 0.011X_1_

Note: a_, l_, and f_ indicate the aril, leaflet, and fruit surface, respectively, the same as below.

**Table 4:** Blocks, treatments, and covariates with significant differences based on the main effect test (*p* < 0.05).

Dependent Variable	Source	*P*	*R* ^2^	Dependent Variable	Source	*P*	*R* ^2^
* a_IAA	** Block	0.005	0.937	* a_ABA	* Block	0.012	0.892
	Treatment	0.231			Treatment	0.691	
** a_IAA-Asp	** Block	0.001	0.977	* a_ABA/ACC	* Block	0.011	0.909
	* Treatment	0.014			Treatment	0.192	
** a_IAA-Glu	* Block	0.016	0.946	* l_ABA	* Block	0.023	0.908
	** Treatment	0.007			* Treatment	0.036	
** a_OxIAA	** Block	<0.001	0.982	** l_TRP	** Block	0.002	0.961
	Treatment	0.054			Treatment	0.121	
a_GA_20_	Block	0.181	0.888	** a_IAA/ABA	* Block	0.025	0.979
	Treatment	0.468			* Treatment	0.015	
	* f_L*	0.043			** FSR	0.008	

Note: The indicators marked with * are dependent variables, fixed variables or covariates with significant test effects. * indicates significant differences (*P* < 0.05) and ** indicates highly significant differences (*P* < 0.01).

**Table 5:** Comparison of the estimated marginal means of the endogenous hormone contents in fruits and FB leaflets for each combination at different levels of block and treatment.

Factor	Level	a_IAA	a_ABA	a_IAA-Asp	a_IAA-Glu	a_OxIAA	a_ABA/ACC	a_IAA/ABA (×10^−3^)	l_ABA	l_TRP (×10^4^)
Block (IFBR)	30	0.451	618	68.833	34.533	47.333	7.681	2.135	67.9	3.737
	60	0.603	555.667	83.467	32.900	51.000	5.856	0	100.367	2.807
	90	0.784	917.667	123.667	40.933	118.333	10.611	1.102	117.567	4.087
Treatment (INFC)	30–40	0.561	713.333	76.800	30.900	62.433	8.121	1.596	86.867	3.686
	60–80	0.627	716.333	97.933	36.033	71.533	8.930	0.366	78.867	3.617
	100–120	0.651	661.667	101.233	41.433	82.700	7.097	0.741	120.100	3.326

**Table 6:** Combinations of the test block and treatment (block × treatment).

Treatment and Levels (INFC)	Block and Levels (IFBR)		
	Block 1 (P-1, 30%)	Block 2 (P-2, 60%)	Block 3 (P-3, 90%)
1 (30–40∙cluster^−1^) (Average 37)	H11	H21	H31
2 (60–80∙cluster^−1^) (Average 75)	H12	H22	H32
3 (100–120∙cluster^−1^) (Average 116)	H13	H23	H33

Note: IFBR, initial fruiting branch rate; INFC, initial number of fruits per cluster.

## Data Availability

The original contributions presented in this study are included in the article. Further inquiries can be directed to the corresponding authors.

## Abbreviations

The following abbreviations are used in this manuscript:

INFC	Initial fruit number per cluster
IFBR	Initial fruiting branch rate
FB	Fruiting branch
FSR	Fruit-shedding rate
TVR	Tree vigor
NRSC	Number of remaining fruits in the single cluster
TNLC	Total number of leaflets in a single cluster
LGS	Leaflet glossiness
MSF	Mass of a single fruit
EPRF	Edible portion rate in a single fruit
TSS	Total soluble solid content
